# Combating the Sigatoka Disease Complex on Banana

**DOI:** 10.1371/journal.pgen.1006234

**Published:** 2016-08-11

**Authors:** Timothy L. Friesen

**Affiliations:** Cereal Crops Research Unit, United States Department of Agriculture – Agricultural Research Service, Fargo, North Dakota, United States of America; Virginia Tech, UNITED STATES

Banana is the fourth most important staple food in the world, behind rice, wheat, and maize, with more than 100 million tons produced annually [1]. Although the majority of bananas produced are consumed locally, banana export is a multi-billion–dollar business [2]. Bananas are grown in more than 100 countries worldwide, largely in developing countries in tropical regions of Africa, Asia, and Latin America [1].

Black Sigatoka disease of banana is one of, if not the most, devastating disease of banana leaves [2]. Black Sigatoka disease is caused by the ascomycete fungus *Pseudocercospora fijiensis*, and this pathogen is part of the larger Sigatoka disease complex that is made up of *P*. *fijiensis*, *P*. *musae* (causal agent of Yellow Sigatoka disease), and *P*. *eumusae* (causal agent of eumusae leaf spot). Management of Black Sigatoka and the Sigatoka complex in general is currently almost completely dependent on frequent (weekly) fungicide treatments throughout the growing season (Fig 1) [3]. Arias et al. [4] estimated the cost of fungicide control at US$1,000/ha for large plantations. Such frequent application of fungicide control of these pathogens has significant socioeconomic impact that includes both environmental and human health hazards [5]. Additionally, the heavy use of fungicides as the primary means of disease management has resulted in resistance to several fungicide classes, reducing efficacy through the development of fungicide-resistant strains [2]. The levels of fungicide treatment used to control the Sigatoka disease complex is not a sustainable practice; therefore, other control solutions are desperately needed.

**Fig 1:**
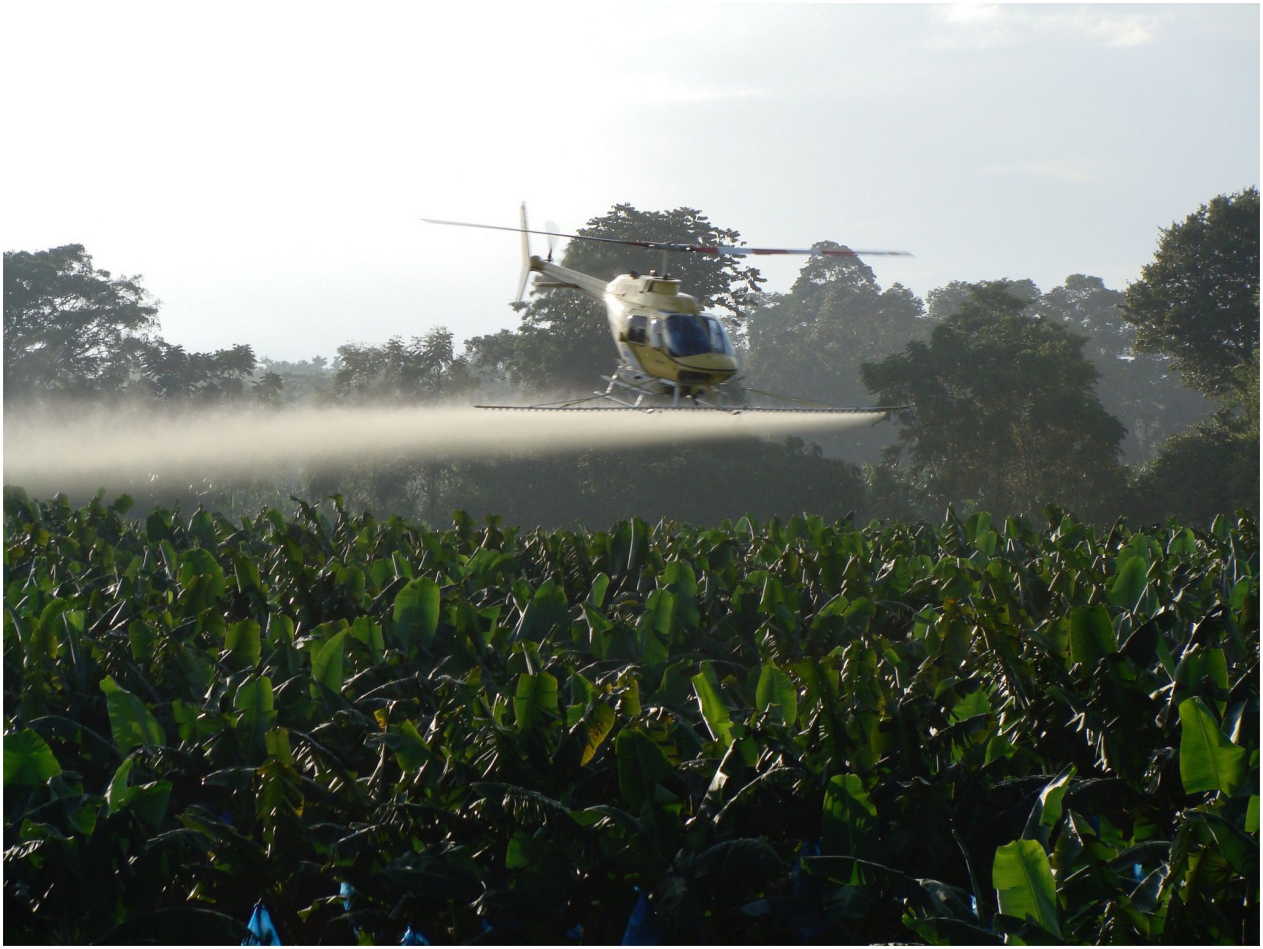
A spray helicopter completing a weekly fungicide application on a banana field affected by the Sigatoka disease complex in Costa Rica. As many as 50 fungicide applications can be made in a year, greatly increasing the risk of fungicide resistance in banana pathogens (photo by Gert Kema).

Several labs from around the world have been working in collaboration to develop tools that are critical to combatting the Sigatoka disease complex. In the current issue, Arango and colleagues have sequenced, assembled, and begun to characterize the genome of *P*. *fijiensis* using full genome sequencing of two different *P*. *fijiensis* isolates [6]. The resulting 74 Mb assembly is currently one of the largest genomes sequenced in the Dothideomycete class, and Arango et al. have shown that this size is primarily due to the genome being highly expanded by long terminal repeat (LTR) retrotransposons. Due to the size of the genome compared to other phylogenetically related pathogens, Arango et al. have suggested that *P*. *fijiensis* and other banana pathogens may have undergone a genome expansion similar to, but independent of, that of the tomato pathogen *Cladosporium fulvum* [7]. The *P*. *fijiensis* genome also shows distinct differences in certain gene families compared to closely related necrotrophic pathogens such as *Parastagonospora nodorum* [8] and *Pyrenophora tritic-repentis* [9]. These gene families include several polyketide synthase and cell wall degrading enzymes that are likely beneficial to the necrotrophic lifestyle but detrimental to the stealth-like biotrophic phase of hemibiotrophic infection employed by *P*. *fijiensis*.

One of the most interesting discoveries to come out of the Arango et al. paper is the discovery of a homolog of the *CfAvr4* gene that was originally discovered in the closely related Dothideomycete pathogen *C*. *fulvum* [6]. CfAvr4 [10] is an effector that binds fungal chitin as a protection mechanism from host chitinases but is also involved in triggering a defense response in tomato plants harboring tomato *Cf4* resistance [11]. Arango et al. not only discovered a *PfAvr4* homolog, but also the likelihood of a corresponding banana resistance gene in the resistant banana cultivar Calcutta 4. The PfAvr4 protein was functionally analyzed by infiltrating purified PfAvr4 into leaves of Calcutta 4, resulting in a hypersensitive-like response similar to what is seen in the *C*. *fulvum*–tomato Avr4–Cf4 interaction. The discovery of an Avr4 homolog that triggers a hypersensitive response in resistant banana provides hope for effective host resistance. The sequencing of these two isolates has laid a strong foundation for future studies investigating the Sigatoka disease complex on banana.

Using the results generated by Arango et al., Chang et al. performed a comparative analysis of *P*. *fijiensis*, *P*. *eumusae*, *and P*. *musae* [12]. These pathogens are known to have evolved on banana from a common ancestor and have obvious differences in virulence, showing independent evolution since speciation. Although the genome sizes of these three pathogens were significantly different, largely due to LTR retrotransposons, total gene content of the three remained similar to other sequenced Dothideomycetes. Interestingly, the more aggressive pathogens in the complex (i.e., *P*. *fijiensis*, *P*. *eumusae*) shared complementary patterns of expansion and contraction of core gene families, suggesting convergent evolution on the banana host. However, small secreted effector-like proteins appear to be very different between all three pathogens, indicating independent host–pathogen evolution.

## Future Prospects

The work presented by Arango et al. and Chang et al. used genome sequencing and comparative analysis to develop a strong foundation for the investigation of each of the species of the Sigatoka disease complex. Future studies can now include functional analysis of the lineage-specific gene families and effectors specific to the Sigatoka complex pathogens. The lineage specificity of these genes indicate the likelihood of these genes/proteins being involved in host–pathogen adaptation with banana. Several genes, especially effector-like genes, were also identified in only one or two of the species. These species-specific effector-like genes are strong candidates for genes being involved in the individual host–pathogen adaptation. Functional characterization of these genes/proteins could lead to the understanding of host targets and, therefore, novel control strategies. Work presented by both Arango et al. and Chang et al. have together laid a strong foundation for future work, providing information and testable hypotheses for generating solutions to this devastating banana disease complex that hitherto was virtually untouched, despite the large economic value of the crop as a staple food for millions of people and as the global top fruit.
